# Two new species of Amanitasect.Phalloideae from Africa, one of which is devoid of amatoxins and phallotoxins

**DOI:** 10.3897/mycokeys.53.34560

**Published:** 2019-06-06

**Authors:** André Fraiture, Mario Amalfi, Olivier Raspé, Ertugrul Kaya, Ilgaz Akata, Jérôme Degreef

**Affiliations:** 1 Meise Botanic Garden, 38 Nieuwelaan, 1860 Meise, Belgium Meise Botanic Garden Meise Belgium; 2 Fédération Wallonie-Bruxelles, Service Général de l’Enseignement supérieur et de la recherche scientifique, 1080 Brussels, Belgium Fédération Wallonie-Bruxelles, Service Général de l’Enseignement supérieur et de la recherche scientifique Brussels Belgium; 3 Duzce University, Faculty of Medicine, Department of Pharmacology, Düzce, Turkey Duzce University Düzce Turkey; 4 Ankara University, Faculty of Science, Department of Biology, Ankara, Turkey Ankara University Ankara Turkey

**Keywords:** Ectomycorrhizal fungi, *
Amanita
*, phylogeny, taxonomy, mycotoxins, tropical Africa, 2 new species

## Abstract

Two new species of Amanitasect.Phalloideae are described from tropical Africa (incl. Madagascar) based on both morphological and molecular (DNA sequence) data. *Amanitabweyeyensis***sp. nov.** was collected, associated with *Eucalyptus*, in Rwanda, Burundi and Tanzania. It is consumed by local people and chemical analyses showed the absence of amatoxins and phallotoxins in the basidiomata. Surprisingly, molecular analysis performed on the same specimens nevertheless demonstrated the presence of the gene sequence encoding for the phallotoxin phallacidin (PHA gene, member of the MSDIN family). The second species, *Amanitaharkoneniana***sp. nov.** was collected in Tanzania and Madagascar. It is also characterised by a complete PHA gene sequence and is suspected to be deadly poisonous. Both species clustered together in a well-supported terminal clade in multilocus phylogenetic inferences (including nuclear ribosomal partial LSU and ITS-5.8S, partial *tef1*-α, *rpb2* and β-tubulin genes), considered either individually or concatenated. This, along with the occurrence of other species in sub-Saharan Africa and their phylogenetic relationships, are briefly discussed. Macro- and microscopic descriptions, as well as pictures and line drawings, are presented for both species. An identification key to the African and Madagascan species of Amanitasect.Phalloideae is provided. The differences between the two new species and the closest *Phalloideae* species are discussed.

## Introduction

Most representatives of Amanitasect.Phalloideae (Fr.) Quél. are famous worldwide for their high, often deadly, toxicity. Currently, the sectionPhalloideae comprises nearly 60 described species, a number of which were described only recently, mainly from Asia (Li et al. 2015, Cai et al. 2016, Thongbai et al. 2017). Moreover, based on a multigene analysis and morphological data, Cai et al. (2014) identified 14 phylogenetic clades potentially representing new species. The phylogenetic analyses made by those authors also resulted in the transfer of several species from sect. Phalloideae to sect. Lepidella Corner & Bas and conversely.

Most of African mycodiversity remains under-explored with only ca. 1500 taxa described to date (Degreef 2018). Very few species belonging to sect. Phalloideae have been recorded from Africa and Madagascar (Walleyn and Verbeken 1998, Tulloss and Possiel 2017). The three poorly known *Amanitaalliiodora* Pat., *A.murinacea* Pat. and *A.thejoleuca* Pat. were described from Madagascar, while *Amanitastrophiolata* Beeli was described from DR Congo (we agree with Gilbert 1941:313 that the var. bingensis Beeli has no taxonomic value). The latter species is the only *Phalloideae* known from Central Africa, together with some doubtful mentions of the imported *A.phalloides* (Fr.: Fr.) Link. *Amanitaphalloides* is only native to Europe, North Africa, Turkey (Kaya et al. 2013, 2015), a certain proportion of the Asian part of Russia and perhaps the West Coast of North America (Pringle and Vellinga 2006, Pringle et al. 2009, Wolfe et al. 2010). Mentions of the species in other regions of the world correspond to either introductions or misidentifications. The exact identity of *A.capensis* and its possible co-specificity with *A.phalloides* remain uncertain. The last species of *Phalloideae* known to Africa, *Amanitamarmorata* Cleland & E.-J. Gilbert (syn.: A.marmoratasubsp.myrtacearum O.K.Mill., Hemmes & G.Wong, *A.reidii* Eicker & Greuning, A.phalloidesf.umbrina ss. African auct.), is also an introduced species. It was described from Australia, growing in association mainly with various species of *Eucalyptus* (e.g. *E.cephalocarpa* Blakely) and subsequently observed in South Africa under *Eucalyptuscloeziana* F.Muell. and *E.* sp. (Eicker et al. 1993, van der Westhuizen and Eicker 1994) and in Hawaii, under *Eucalyptusrobusta* Sm., *E.saligna* Sm., *E.* sp., *Araucariacolumnaris* Hook., *Melaleucaquinquenervia* (Cav.) S.T.Blake, as well as under pure *Casuarinaequisetifolia* L. (Miller et al. 1996).

Amatoxins and phallotoxins are responsible for the high toxicity of Amanitasect.Phalloideae. Nevertheless, apart from *Amanitaalliiodora*, considered toxic by the Madagascan people, and the deadly poisonous *A.phalloides* (incl. “*A.capensis*”) and probably *A.marmorata*, no data are available attesting to the toxicity or the edibility of the Madagascan and African species.

In the framework of taxonomic and phylogenetic studies of Amanitasect.Phalloideae, specimens originating from tropical Africa were critically studied. Morphological and multigenic phylogenetic studies proved to be concordant and established the existence of two distinct species that could not be identified as any known taxa. *Amanitabweyeyensis* from the western province of Rwanda and *A.harkoneniana* from the Tanzanian Miombo woodlands and Madagascar are described here as new. Their phylogenetic affinities with other *Amanita* species reported from Africa are discussed and a key to African species of Amanitasect.Phalloideae is provided.

## Materials and methods

### Specimens studied

African *Amanitaphalloides*-related specimens held in BR were studied in depth (*Degreef* 653 from Burundi; *Degreef* 1257 and 1304, both from Rwanda). A picture appearing in Härkönen et al. (2003: 62, sub “*Amanita* species which looks very much like *Amanitaphalloides*”) convinced us to also check the specimen *Saarimäki* 591 (from Tanzania). We additionally obtained *Saarimäki* et al. 1061 (also from Tanzania) on loan from the University of Helsinki (H). Finally, P. Pirot sent us two unnumbered specimens he collected in Madagascar in 2014 and 2016.

We also examined for comparison the type specimen of Amanitamarmoratasubsp.myrtacearum (*O.K. Miller* 24545, VPI) collected in Hawaii and 3 specimens of *Amanitamarmorata* collected in Australia: *H.D. Weatherhead* s.n. (= MEL 2028859A) and *J.B. Cleland* s.n. (= AD-C 3083 and 3085). We unsuccessfully tried to obtain the type specimen of *Amanitareidii* on loan. Braam Vanwyk informed us that the holotype preserved in PRU had unfortunately been destroyed and no longer exists. Although Miller et al. (1996: 144) mentioned having received a fragment of that type specimen on loan from PREM, it seems that no such material exists in the collections of that institution (Riana Jacob-Venter, in e-litt.), nor in K (Angela Bond, in e-litt.). We also received on loan the lectotypus of *Amanitamurina* (Cooke & Massee) Sacc. (*Bailey* 651, K, correct name: *Amanitaneomurina* Tulloss).

### Macro- and microscopic studies

Macroscopic characters were deduced from herbarium specimens, as well as from specimen labels, field notes and pictures, when available. Microscopic examinations were carried out using an Olympus BX51 microscope, from herbarium material mounted in ammoniacal Congo Red or in Melzer’s reagent. Measurements were made using a camera lucida and a calibrated scale. In the descriptions, figures between brackets are extreme values, underlined figures are averages, Q values are length/width ratios of spores, l/w values are the same ratios for other types of cells. Mentions like “[60/4/2]” after measurements of spores (or other microscopic structures) mean 60 spores measured, from 4 different basidiomata collected in 2 different places.

### Molecular analyses

#### DNA extraction, amplification and sequencing

Genomic DNA was isolated from CTAB-preserved tissues or dry specimens using a CTAB isolation procedure adapted from Doyle and Doyle (1990). PCR amplification of the ITS region (nuclear ribosomal internal transcribed spacer) and LSU (large subunit ribosomal DNA) was performed using the primer pairs ITS4/ITS5 or ITS1-F/ITS4 and LR0R/LR5, respectively (http://biology.duke.edu/fungi/mycolab/primers.htm). Parts of the protein-coding genes β-tubulin, *rpb2* (second largest subunit of RNA polymerase II) and *tef-1* (translation elongation factor 1 alpha) were amplified using the primer pairs Am-β-tub-F/Am-β-tub-R, Am-6F/Am-7R and EF1-983F/EF1-1567R, respectively (Zhang et al. 2010). PCR products were purified by adding 1 U of Exonuclease I and 0.5 U FastAP Alkaline Phosphatase (Thermo Scientific, St. Leon-Rot, Germany) and incubating at 37 °C for 1 h, followed by inactivation at 80 °C for 15 min.

Sequencing was performed by Macrogen Inc. (Korea and The Netherlands) using the same primer combinations as for PCR, except for Am-β-tub-F, which was replaced by the shorter primer Am-β-tub-F-Seq (5’-CGGAGCRGGTAACAAYTG-3’) following Thongbai et al. (2017). The sequences were assembled in Geneious Pro v. 6.0.6 (Biomatters).

#### Phylogenetic analysis

Thirty-five sequences of *Amanita* specimens were newly generated for this study and deposited in GenBank (http://www.ncbi.nlm.nih.gov/; Table 1). Initial BLAST searches (http://blast.ncbi.nlm.nih.gov) of both LSU and ITS-5.8S sequences were performed to estimate similarity with *Amanita* sequences already present in Genbank database (Table 1). Additional sequences were selected from previously published phylogenies and from GenBank (Table 1). The quality of the sequences was taken into account in selecting the sequences for the phylogenetic analyses. Materials and sequences used in this study are listed in Table 1.

**Table 1.** List of collections used for DNA analyses, with origin, GenBank accession numbers and references.

Species		GenBank accession no.				
Specimen voucher	Country	LSU	ITS	*rpb2*	*tef1*–α	βtubulin
Sect. Phalloideae						
*Amanitaalliodora* Pat. 1928						
DSN062	Madagascar	KX185612	KX185611	–	–	–
*Amanita amerivirosa nom. prov.*						
RET 397-8	USA	KJ466460	KJ466398	–	KJ481964	KJ466543
RET 480-1	USA	KJ466461	KJ466399	KJ466630	KJ481965	KJ466544
*Amanitabisporigera* G.F. Atk. 1906						
RET 377-9	USA	KJ466434	KJ466374	–	KJ481936	KJ466501
*Amanitabrunneitoxicaria* Thongbai, Raspé& K.D. Hyde 2017						
BZ2015-01	Thailand	–	NR_151655	KY656879	–	KY656860
*Amanitabweyeyensis* Fraiture, Raspé & Degreef, sp. nov.						
clone Agar_8B_S114	Madagascar	–	KT200567	–	–	–
JD 1257	Rwanda	MK570926	MK570919	–	–	–
JD 1304	Rwanda	MK570927	MK570920	MK570931	MK570940	MK570916
TS 591	Tanzania	MK570928	MK570921	–	–	–
*Amanitadjarilmari* E.M. Davison 2017						
EMD 008 cl_4	Australia	–	KU057382	–	–	–
EMD 008 cl_5	Australia	–	KU057383	–	–	–
EMD 008 cl_6	Australia	–	KU057384	–	–	–
EMD 5 010 l_1	Australia	–	KU057393	–	–	–
EMD 5 010 l_15	Australia	–	KU057392	–	–	–
EMD 5 010 l_3	Australia	–	KU057391	–	–	–
EMD 5 010 l_5	Australia	–	KU057390	–	–	–
EMD 5 010 l_7	Australia	–	KU057389	–	–	–
EMD 8 013 l_1	Australia	–	KU057399	–	–	–
EMD 8 013 l_2	Australia	–	KU057400	–	–	–
EMD 8 013 l_3	Australia	–	KU057401	–	–	–
EMD 8 013 l_4	Australia	–	KU057402	–	–	–
EMD 8 013 l_5	Australia	–	KU057403	–	–	–
PERTH08776040	Australia	KY977708	–	–	MF037234	MF000743
PERTH08776067 l_1	Australia	KY977704	KY977732	MF000755	MF000750	MF000742
PERTH08776067 l_2	Australia	KY977704	KY977733	MF000755	MF000750	MF000742
PERTH08776067 l_3	Australia	KY977704	KY977734	MF000755	MF000750	MF000742
PERTH08776067 l_4	Australia	KY977704	KY977735	MF000755	MF000750	MF000742
PERTH08776067 l_5	Australia	KY977704	KY977736	MF000755	MF000750	MF000742
PERTH08776075 l_1	Australia	KY977706	KY977737	–	–	–
PERTH08776075 l_2	Australia	KY977706	KY977738	–	–	–
PERTH08776075 l_3	Australia	KY977706	KY977739	–	–	–
PERTH08776075 l_4	Australia	KY977706	KY977740	–	–	–
PERTH08776075 l_5	Australia	KY977706	KY977741	–	–	–
PERTH08776083 l_1	Australia	KY977710	KY977742	–	–	MF000744
PERTH08776083 l_2	Australia	KY977710	KY977743	–	–	MF000744
PERTH08776083 l_3	Australia	KY977710	KY977744	–	–	MF000744
PERTH08776083 l_4	Australia	KY977710	KY977745	–	–	MF000744
PERTH08776083 l_5	Australia	KY977710	KY977746	–	–	MF000744
*Amanitaeucalypti* O.K. Mill. 1992						
PERTH8809828 cl_3	Australia	KY977707	KU057380	MF000758	MF000751	MF000746
PERTH8809828 cl_4	Australia	KY977707	KU057397	MF000758	MF000751	MF000746
PERTH8809828 cl_5	Australia	KY977707	KU057396	MF000758	MF000751	MF000746
PERTH8809828 cl_6	Australia	KY977707	KU057395	MF000758	MF000751	MF000746
PERTH8809828 cl_7	Australia	KY977707	KU057394	MF000758	MF000751	MF000746
PERTH8809828 l_2	Australia	KY977707	KU057398	MF000758	MF000751	MF000746
PERTH8809968 cl_3	Australia	KY977707	KU057380	MF000758	MF000751	MF000746
PERTH8809968 cl_4	Australia	KY977707	KU057381	MF000758	MF000751	MF000746
PERTH8809828 cl_1	Australia	KY977707	KU057398	MF000758	MF000751	MF000746
*Amanitaexitialis* Zhu L. Yang & T.H. Li 2001						
HKAS74673	China	KJ466435	KJ466375	KJ466590	KJ481937	KJ466502
HKAS75774	China	JX998052	JX998027	KJ466591	JX998001	KJ466503
HKAS75775	China	JX998053	JX998026	KJ466592	JX998002	KJ466504
HKAS75776	China	JX998051	JX998025	KJ466593	JX998003	KJ466505
*Amanitafuliginea* Hongo 1953						
HKAS75780	China	JX998048	JX998023	KJ466595	JX997995	KJ466507
HKAS75781	China	JX998050	JX998021	KJ466596	JX997994	KJ466508
HKAS75782	China	JX998049	JX998022	KJ466597	JX997996	KJ466509
HKAS77132	China	KJ466436	KJ466375	KJ466598	KJ481939	KJ466510
HKAS79685	China	KJ466437	KJ466376	KJ466594	KJ481938	KJ466506
*Amanitafuligineoides* P. Zhang & Zhu L. Yang 2010						
HKAS52727	China	JX998047	JX998024	KJ466599	–	KJ466511
LHJ140722-13	China	KP691685	KP691696	KP691705	KP691674	KP691715
LHJ140722-18	China	KP691686	KP691697	KP691706	KP691675	KP691716
*Amanitagardneri* E.M. Davison 2017						
EMD 8-2010 cl_1	Australia	–	KU057387	–	–	–
EMD 8-2010 cl_3	Australia	–	KU057388	–	–	–
EMD 8-2010 cl_4	Australia	–	KU057386	–	–	–
EMD 8-2010 cl_6	Australia	–	KU057385	–	–	–
PERTH08776121	Australia	KY977712	–	MF000756	MF000752	MF000748
*Amanitagriseorosea* Q. Cai, Zhu L. Yang & Y.Y. Cui 2016						
HKAS77334	China	KJ466476	KJ466413	KJ466661	KJ481994	KJ466580
HKAS77333	China	KJ466475	KJ466412	KJ466660	KJ481993	KJ466579
*Amanitaharkoneniana* Fraiture & Saarimäi, sp. nov.						
P Pirot SN	Madagascar	MK570929	MK570922	MK570938	MK570941	MK570917
TS 1061	Tanzania	MK570930	MK570923	–	–	–
*Amanitamarmorata* Cleland & E.-J. Gilbert 1941						
HW N	Australia	MK570931	MK570924	MK570939	MK570942	MK570918
PERTH 8690596 cl_1	Australia	KY977711	KU057408	–	–	MF000749
PERTH 8690596 cl_2	Australia	KY977711	KU057404	–	–	MF000749
PERTH 8690596 cl_3	Australia	KY977711	KU057405	–	–	MF000749
PERTH 8690596 cl_4	Australia	KY977711	KU057406	–	–	MF000749
PERTH 8690596 cl_5	Australia	KY977711	KU057407	–	–	MF000749
RET 623-7	Australia	KP757874	KP757875	–	–	–
RET 85-9	Australia	MG252697	MG252696	–	–	–
*Amanitamarmoratasubsp.myrtacearum* O.K. Mill., Hemmes & G. Wong 1996						
DED 5845	Hawai	AY325881	AY325826	–	–	–
*Amanitamillsii* E.M. Davison & G.M. Gates 2017						
HKAS77322	Australia	KJ466457	KJ466395	KJ466643	KJ481978	KJ466557
HO581533 l_2	Australia	KY977713	KY977715	MF000753	MF000759	MF000760
HO581533 l_1	Australia	KY977713	KY977714	MF000753	MF000759	MF000760
HO581533 l_3	Australia	KY977713	KY977716	MF000753	MF000759	MF000760
HO581533 l_5	Australia	KY977713	KY977717	MF000753	MF000759	MF000760
*Amanitamolliuscula* Q. Cai, Zhu L. Yang & Y.Y. Cui 2016						
HKAS75555	China	KJ466471	KJ466408	KJ466638	KJ481973	KJ466552
HMJAU20469	China	KJ466473	KJ466410	KJ466640	KJ481975	KJ466554
HKAS77324	China	NG_057038	NR_147633	KJ466639	KJ481974	KJ466553
*Amanitaocreata* Peck 1909						
HKAS79686	USA	KJ466442	KJ466381	KJ466607	KJ481947	KJ466518
*Amanitapallidorosea* P. Zhang & Zhu L. Yang 2010						
HKAS61937	China	KJ466443	KJ466382	KJ466609	KJ481949	KJ466520
HKAS71023	Japan	KJ466444	KJ466383	KJ466624	KJ481960	KJ466536
HKAS75483	China	KJ466445	KJ466384	KJ466623	KJ481959	KJ466535
HKAS75783	China	JX998055	JX998035	KJ466625	JX998010	KJ466537
HKAS75784	China	JX998056	JX998036	KJ466626	JX998009	KJ466538
HKAS75786	China	JX998054	JX998037	KJ466627	JX998011	KJ466539
HKAS77329	China	KJ466447	KJ466387	KJ466610	KJ481950	KJ466521
HKAS77348	China	KJ466448	KJ466387	KJ466611	KJ481951	KJ466522
HKAS77349	China	KJ466449	KJ466389	KJ466628	KJ481961	KJ466540
HKAS77327	China	KJ466446	KJ466386	KJ466608	KJ481948	KJ466519
*Amanitaparviexitialis* Q. Cai, Zhu L. Yang & Y.Y. Cui 2016						
HKAS79049	China	NG_057092	–	KT971345	KT971343	KT971346
*Amanitaphalloides* Secr. 1833						
HKAS75773	USA	JX998060	JX998031	KJ466612	JX998000	KJ466523
*Amanitarimosa* P. Zhang & Zhu L. Yang 2010						
HKAS75778	China	JX998045	JX998019	KJ466616	JX998006	KJ466527
HKAS75779	China	JX998046	JX998020	KJ466617	JX998004	KJ466528
HKAS77105	China	KJ466452	KJ466391	KJ466618	KJ481954	KJ466529
HKAS77120	China	KJ466453	KF479044	KJ466619	KJ481955	KJ466530
HKAS77279	China	KJ466454	KJ466392	KJ466620	KJ481956	KJ466531
HKAS77335	China	KJ466455	KJ466393	KJ466621	KJ481957	KJ466532
HKAS77336	China	KJ466456	KJ466394	KJ466622	KJ481958	KJ466533
HKAS75777	China	JX998044	JX998018	KJ466615	JX998005	KJ466526
*Amanita* sp. 10 ZLY2014						
HKAS77322	Australia	KJ466457	KJ466395	KJ466643	KJ481978	KJ466557
*Amanita* sp. 2 ZLY2014						
HKAS77350	China	KJ466462	KJ466400	KJ466631	KJ481966	KJ466545
*Amanita* sp. 3 ZLY2014						
HKAS77342	China	KJ466463	KF479045	KJ466632	KJ481967	KJ466546
HKAS77343	China	KJ466464	KJ466401	KJ466633	KJ481968	KJ466547
HKAS77344	China	KJ466465	KJ466402	KJ466634	KJ481969	KJ466548
HKAS77351	China	KJ466466	KJ466403	KJ466635	KJ481970	KJ466549
*Amanita* sp. 5 ZLY2014						
RET 422-8	USA	KJ466469	KJ466406	KJ466649	KJ481983	KJ466563
RET 493-6	USA	KJ466470	KJ466407	KJ466650	KJ481984	KJ466564
*Amanita* sp. 8 ZLY2014						
HKAS75150	Bangladesh	KJ466477	KJ466414	KJ466641	KJ481976	KJ466555
*Amanita* sp. 9 ZLY2014						
HKAS77323	China	KJ466478	KJ466415	KJ466642	KJ481977	KJ466556
*Amanitasuballiacea* (Murrill) Murrill 1941						
RET 490-1	USA	KJ466485	KJ466420	KJ466601	KJ481941	KJ466513
RET 491-7	USA	KJ466486	KJ466421	KJ466602	KJ481942	KJ466514
RET 478-6	USA	KJ466484	KJ466419	KJ466600	KJ481940	KJ466512
*Amanitasubfuliginea* Q. Cai, Zhu L. Yang & Y.Y. Cui 2016						
HKAS77347	China	KJ466468	KJ466405	KJ466637	KJ481972	KJ466551
HKAS77326	China	KJ466467	KJ466404	KJ466636	KJ481971	KJ466550
*Amanitasubjunquillea* S. Imai 1933						
HKAS74993	China	KJ466489	KJ466424	KJ466652	KJ481987	KJ466570
HKAS75770	China	JX998062	JX998034	KJ466653	JX997999	KJ466571
HKAS75771	China	JX998063	JX998032	KJ466654	JX997997	KJ466572
HKAS75772	China	JX998061	JX998033	KJ466655	JX997998	KJ466573
HKAS77325	China	KJ466490	KJ466425	KJ466656	KJ481988	KJ466574
HKAS77345	China	KJ466491	KJ466426	KJ466657	KJ481989	KJ466575
HMJAU20412	China	KJ466492	KJ466427	KJ466658	KJ481990	KJ466576
HMJAU23276	China	KJ466493	KJ466428	KJ466659	KJ481991	KJ466577
HKAS63418	China	KJ466488	KJ466423	KJ466651	KJ481986	KJ466569
*Amanitasubpallidorosea* Hai J. Li 2015						
LHJ140923--41	China	KP691692	KP691683	KP691701	KP691670	KP691711
LHJ140923-55	China	KP691693	KP691680	KP691702	KP691671	KP691712
LHJ140923-17	China	KP691691	KP691677	KP691700	KP691669	KP691713
*Amanitavirosa* Secr. 1833						
HKAS71040	Japan	KJ466496	KJ466429	KJ466665	KJ481997	KJ466584
HMJAU20396	China	JX998059	JX998029	–	JX998008	KJ466585
HMJAU23303	China	KJ466497	KJ466430	KJ466666	KJ481998	KJ466586
HMJAU23304	China	KJ466498	KJ466431	KJ466667	KJ481999	KJ466587
HKAS56694	Finland	JX998058	JX998030	KJ466664	JX998007	KJ466583
*Amanitahalloides ar lba* Costantin & L.M. Dufour 1895						
AF2322	Belgium	–	MK570925	–	–	–
*Amanitahalloides ar mbrina* (Ferry) Maire 1937						
PREM 48618	South Africa	AY325882	AY325825	–	–	–
*Amanitaeidii* Eicker & Greuning 1993						
PRU 4306	South Africa	AY325883	AY325824	–	–	–
*Amanita* p						
CM13 09	New Caledonia	–	KY774002	–	–	–
*Amanita* p Kerala01						
RET 91-7	India	–	KC855219	–	–	–
*Incertae sedis*						
*Amanitaballerina* Raspé Thongbai & K.D. Hyde 2017						
OR1014	Thailand	–	KY747466	KY656883	–	KY656864.
OR1026	Thailand	MH157079	KY747467	KY656884	–	KY656865
*Amanitafranzii* Zhu L. Yang, Y.Y. Cui & Q. Cai 201						
HKAS77321	China	KJ466481	MH508357	KJ466646	MH508798	KJ466560
HKAS91231	China	MH486525	MH508358	MH485994	MH508801	MH485516
*Amanitapseudogemmata* Hongo 1974						
HKAS85889	China	MH486768	–	MH486186	MH508995	MH485692
HKAS84744	China	MH486767	–	MH486185	MH508994	MH485691
*Amanitazangii* Zhu L. Yang, T.H. Li & X.L. Wu 2001						
GDGM29241	China	KJ466499	KJ466432	KJ466668	KJ482000	KJ466588
HKAS77331	China	KJ466500	KJ466433	KJ466669	KJ482001	KJ466589
Sect. Validae						
*Amanitacfspissacea* S. Imai 1933						
OR1214	Thailand	KY747478	KY747469	KY656886	–	KY656867

Note: cl_ stands for clone. References to sequences retrieved from GenBank: Cai et al. (2012), Cai et al. (2014), Cui et al. (2018), Davison et al. (2017), Henry et al. (2015), Houles et al. (2018), Li et al. (2015), Thongbai et al. 2017, Tulloss (continuously updated).

A combined dataset (including nuclear ribosomal partial LSU and ITS-5.8S, partial *tef1*-α, *rpb2* and β-tubulin genes), comprising sequences from 94 collections including the outgroup and an ITS-5.8S / LSU dataset of 69 sequences, including several clones derived from the same collections and the outgroup, were constructed and used for further phylogenetic analyses.

Amanitacf.spissacea voucher OR1214 and *Amanitasubjunquillea* voucher HKAS63418 were used as outgroups for the combined and ITS-LSU datasets, respectively (Thongbai et al. 2017, Cui et al. 2018).

Nucleotide sequences were automatically aligned using the MUSCLE algorithm (Edgar 2004) with default settings. The alignment was further optimised and manually adjusted as necessary by direct examination with the software Se-Al v. 2.0a11 (University of Oxford).

The assignment of codon positions in the protein-coding sequences was confirmed by translating nucleotide sequences into predicted amino acid sequences using MacClade 4.0 (Maddison and Maddison 2000) and then compared with the annotated *Amanitabrunnescens* sequences AFTOL-ID 673.

Potential ambiguously aligned segments, especially in the three introns present in *tef-1* and β-tubulin gene sequences and in the ITS-5.8S alignment, were detected by Gblocks v0.91b (Castresana 2000; http://molevol.cmima.csic.es/castresana/Gblocks.html) with the following parameter settings: minimum number of sequences for a conserved position = 24 (minimum possible); minimum number of sequences for a flank position = 24 (minimum possible); maximum number of contiguous non-conserved positions = 4 bp, minimum block size = 4 bp and gaps allowed within selected blocks in half of the sequences.

To detect the possible bias from substitution saturation and to evaluate the phylogenetic signal, we tested each partition of the combined dataset and the ITS-LSU dataset by using Xia’s test (Xia et al. 2003, Xia and Lemey 2009), as implemented in DAMBE (Xia and Xie 2001). As the Iss.c is based on simulation results, there is a problem with more than 32 species. To circumvent this problem, DAMBE was used to randomly sample subsets of 4, 8, 16 and 32 OTUs multiple times and to perform the test for each subset to see if substitution saturation exists for these subsets of sequences. In order to confirm the results of the Xia’s method, we also plotted the raw number of transversions and transitions against Tamura-Nei genetic distances with the aid of the DAMBE package, with an asymptotic relationship indicating the presence of saturation.

Models of evolution for BI were estimated using the Akaike Information Criterion (AIC) as implemented in Modeltest 3.7 (Posada and Crandall 1998).

The dataset was subdivided into 10 data partitions: *tef-1* 1^st^ and -2^nd^ codon positions, *tef-1* -3^rd^ codon positions, *tef-1* introns and *rpb2* 1^st^ and -2^nd^ codon positions, *rpb2* -3^rd^ codon positions, β-tubulin 1^st^ and -2^nd^ codon positions, β-tubulin -3^rd^ codon positions, β-tubulin intron, ITS, LSU. Phylogenetic analyses were performed separately for each individual and concatenated loci using Bayesian Inference (BI) as implemented in MrBayes v3. 2 (Ronquist et al. 2012) and Maximum Likelihood (ML) as implemented in RAxML 7.2.7 (Stamatakis et al. 2008).

The best-fit models for each partition were implemented as partition specific models within partitioned mixed-model analyses of the combined dataset (Table 2). All parameters were unlinked across partitions. Bayesian analyses were implemented with two independent runs, each with four simultaneous independent chains for ten million generations, starting from random trees and keeping one tree every 1000th generation. All trees sampled after convergence (average standard deviation of split frequencies < 0.01 and confirmed using Tracer v1.4 [Rambaut and Drummond 2007]) were used to reconstruct a 50% majority-rule consensus tree (BC) and to calculate Bayesian Posterior Probabilities (BPP). BPP of each node was estimated based on the frequency at which the node was resolved amongst the sampled trees with the consensus option of 50% majority-rule (Simmons et al. 2004). A probability of 0.95 was considered significant. Maximum Likelihood (ML) searches conducted with RAxML involved 1000 replicates under the GTRGAMMAI model, with all model parameters estimated by the programme. In addition, 1000 bootstrap (ML BS) replicates were run with the same GTRGAMMAI model. We provided an additional alignment partition file to force RAxML software to search for a separate evolution model for each dataset. Clades with Maximum Likelihood bootstrap values of 75% or greater were considered supported by the data.

**Table 2.** Summary of data sets of ITS rDNA, nuc-LSU rDNA, *tef1*-α, *rpb2* and β-tubulin.

**Datasets**										
Properties	*tef1* 1^st^ & 2^nd^	*tef1* 3^rd^	*tef1* introns	*rpb2* 1^st^& 2^nd^	*rpb2* 3^rd^	β-tubulin 1^st^& 2^nd^	β-tubulin 3^rd^	β-tubulin introns	nucLSU	ITS
Alignment size	296	147	147	452	226	167	83	171	887	935
Excluded characters	–	–	–	–	–	–	–	–	–	557
Model selected	GTR+I+G	GTR+G	HKY+I	GTR+I	GTR+G	SYM+I+G	HKY+G	HKY+G	GTR+I+G	HKY+I+G
-Likelihood score	780.2892	1857.1256	1535.9010	1285.5159	3033.6099	1319.9380	1108.1555	1023.9042	3403.9714	4844.7563
Base frequencies										
Freq. A =	0.3179	0.1686	0.2479	0.2914	0.2478	Equal	0.1745	0.2254	0.2877	0.3023
Freq. C =	0.2276	0.3231	0.2175	0.2132	0.1956	Equal	0.3113	0.1690	0.1671	0.1846
Freq. G =	0.2536	0.2159	0.1807	0.2761	0.2541	Equal	0.2257	0.2228	0.2937	0.2068
Freq. T =	0.2010	0.2924	0.3540	0.2192	0.3025	Equal	0.2885	0.3827	0.2515	0.3062
Proportion of invariable sites	0.8042	–	0.0940	0.8283	–	0.4975	–	–	0.5726	0.2855
Gamma shape	0.7888	2.1595	-	-	2.7065	4.2837	3.7320	0.8697	0.5839	0.8470
Test of substitution saturation										
Iss	0.263	0.354	0.723	0.335	0.308	0.156	0.306	0.662	0.499	0.472
Iss.cSym	0.683	0.721	0.928	0.697	0.685	0.706	0.875	0.776	0.764	0.707
P (Sym)	< 0.0001	< 0.0001	0.2135	< 0.0001	< 0.0001	< 0.0001	< 0.0001	0.402	< 0.0001	< 0.0001
Iss.cAsym	0.354	0.668	0.802	0.502	0.458	0.407	0.711	0.535	0.675	0.645
P (Asym)	< 0.0001	< 0.0001	0.6284	< 0.0001	< 0.0001	< 0.0001	< 0.0001	0.354	< 0.0001	< 0.0001

Note: Iss: index of substitution saturation. Iss.cSym: critical value for symmetrical tree topology. Iss.cAsym: critical value for extremely assymetrical tree topology. P: probability that Iss is significantly different from the critical value (Iss.cSym or Iss.cAsym).

To detect topological conflicts amongst data partitions, the nodes between the majority-rule consensus trees obtained in the ML analysis from the individual datasets were compared with the software compat.py (available at www.lutzonilab.net/downloads). Paired trees were examined for conflicts only involving nodes with ML BS > 75% (Mason-Gamer and Kellogg 1996, Lutzoni et al. 2004, Reeb et al. 2004). A conflict was assumed to be significant if two different relationships for the same set of taxa (one being monophyletic and the other not) were observed in rival trees. Sequence data and statistical analysis for each individual dataset and combined analysis are provided in Table 2.

#### PCR amplification of *Amanita* toxins genes family members

Two major toxin-encoding genes, AMA1 and PHA1, directly encode for α-amanitin and the related bicyclic heptapeptide phallacidin, the lethal peptide toxins of poisonous mushrooms in the genus *Amanita*. α-Amanitin and phallacidin are synthesised as pro-proteins of 35 and 34 amino acids, respectively, in the ribosomes and are later cleaved by a prolyl oligopeptidase (Hallen et al. 2007, Luo et al. 2009, Li et al. 2014). In these pro-proteins, the amino acid sequences found in the mature toxins are flanked by conserved amino acid sequences, with an invariant Pro residue immediately upstream of the toxin regions and as the last amino acid in the toxin regions.

The toxins genes and MSDIN (cyclic peptide precursor) family members and related sequences were amplified from total genomic DNA with two consecutive PCR reactions, using the products of the first PCR as templates for the second one. For the first PCR, we used degenerated primers forward (5’ATGTCNGAYATYAAYGCNACNCG3’) and the reverse primer (5’CCAAGCCTRAYAWRGTCMACAAC3’), following the cycling condition detailed in Li et al. (2014).

For the nested PCR amplification (using the PCR products above as the amplification template of AMA1 and PHA1 genes), primers targeting conserved regions of MSDINs family were obtained from previous studies (Hallen et al. 2007, Luo et al. 2009, Li et al. 2014, Wołoszyn and Kotłowski 2017) or designed *ad hoc* against the conserved upstream and downstream sequences of AMA1 and PHA1 available on Genbank and tested in different combinations. For α-amanitin, we used 5’CCATCTGGGGCATCGGTTGCAACC3’ as forward primer (Li et al. 2014) in combination with the reverse primers 5’CTACGTYYGAGTCAGGACAACTGCC3’ (Li et al. 2014) and the newly generated AMA-α-R2 (5’GTCAAAGTCAGTGCGACTGCCTTGT3’) and AMA-α-R3 (5’CTGCATTTGAGTTAGGATAACGACA3’). We also tested primer pairs AMAF and AMAR 5 (Wołoszyn and Kotłowski 2017). For β-amanitin, we used forward primer AMA-β-F (5’CCATMTGGGGMATMGGTTGYRACC3’) in combination with reverse primers AMA-β-R (5’GTCMACAACTYGTATYGKCCACTACT3’), AMA-β-R2 (5’GTCMACAACTYRTATYGKCCACMGCT3’) and AMA-β-R3 (5’CCTRAYAWRGTCMACAACT3’). For PHA genes, we used forward primer 5’CCTGCYTGGCTYGTAGAYTGCCCA3’ (Li et al. 2014) in combination with the reverse primers 5’CGTCCACTACTAYDTCMARGTCAGTAC3’ (Li et al. 2014) and AMA-PHA-R2 (5’AGTCACGACTACATCGAGGTCAGTACA3’). Primer pairs FALF and FALR (Wołoszyn and Kotłowski 2017) were also tested for amplification of the phallotoxins genes.

Thermal cycling conditions were: initial denaturation at 94°C for 4 min, followed by 33 cycles of denaturation at 94°C for 30 s, annealing at 59°C for 30 s, extension at 72°C for 30 s and a final extension at 72°C for 7 min, for all reactions except for the ones involving primers from Wołoszyn and Kotłowski (2017), for which an annealing temperature of 68°C for 1 min was used.

### Chemical analyses

#### Mushroom preparation

Two groups of dried mushrooms, i.e. with cuticle (n=3) and without cuticle (n=3), were analysed. For each specimen, 100 mg of dry tissues were ground and homogenised in 3 ml extraction medium (methanol:water:0.01 M HCl [5:4:1, v/v/v]) using a tissue homogeniser. After 1 hour of incubation, all extracts were centrifuged at 5000 rpm for 5 min, the supernatant was filtered using a 0.45 mm syringe filter and 20 µl of this supernatant was injected in the RP-HPLC device for toxin detection.

#### Standard solutions and chemicals

The α-amanitin and phalloidin standards were obtained from Sigma-Aldrich (USA). The β-amanitin, γ-amanitin and phallacidin standards were obtained from Enzo Life Sciences (Farmingdale, NY, USA). The solvents used in this study were all HPLC grade. Stock solutions of all toxins (100 µg/ml) were prepared in methanol. The calibration standards of all toxins were diluted in the extraction fluid in concentrations of 1, 5, 20, 100, 200, 500 ng/ml. Calibration curves were produced for each toxin; they were linear over the range of interest (R^2^ > 0.99).

#### RP-HPLC analysis of toxins

Chromatography conditions for the procedure followed in this study were reported by Kaya et al. (2013, 2015). In short, the authors reported excellent separation of amatoxins and phallotoxins with the RP-HPLC and UV detection. In the laboratory, an RP-HPLC analysis of mushroom extracts was performed on a Shimadzu (Japan) HPLC system. The RP-HPLC analysis of standard solutions of α-amanitin, β-amanitin, γ-amanitin, phalloidin, phallacidin and subsequent quantification of mushroom extracts were performed on 150 × 4.6 mm, 5 mm particle, C18 column (Agilent Technologies, Palo Alto, CA) with 302 nm (for amatoxins) and 290 nm (for phallotoxins) at the UV detector. The mobile phase was used in isocratic profile with a flow rate of 1 ml/min. The content of the mobile phase was 0.05 M ammonium acetate (pH 5.5 with acetic acid)/acetonitrile (90:10 v/v). The detection limits were set at 0.6 ng/g for all toxins.

## Results

### Molecular analyses

#### Phylogenetic analysis

By comparing the tree topologies obtained for the individual datasets, no significant conflict, involving significantly supported nodes, was found using the 75% ML BP criterion; the datasets were therefore combined.

**Figure 1.**
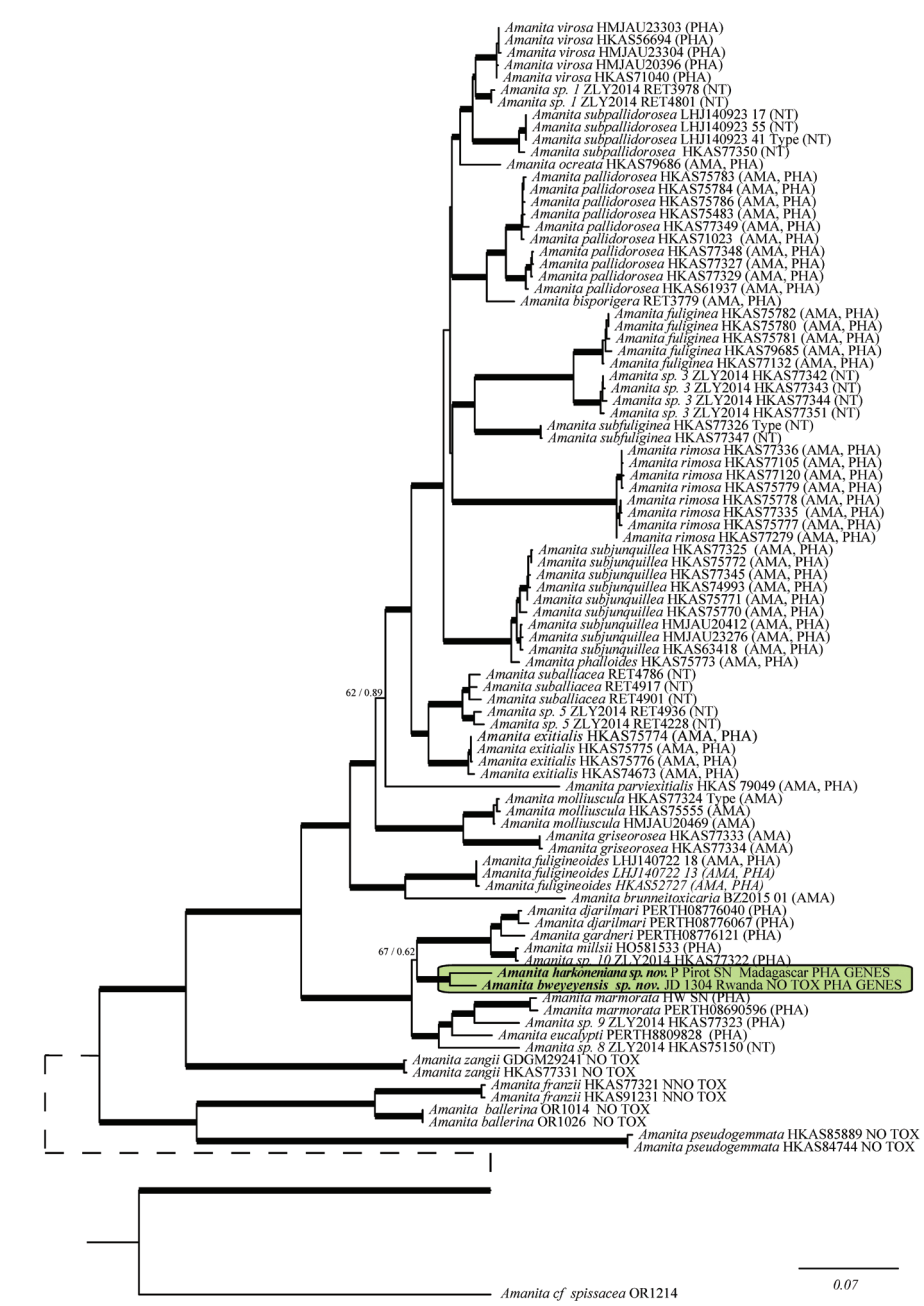
The 50% majority-rule consensus tree from Bayesian inference of the combined dataset. Thickened branches in bold represent ML BS support greater than 75% and BPP greater than 0.95; thickened branches in grey denote branches supported by either ML BS or BPP. For selected nodes ML BS support value and BPP are, respectively, indicated to the left and right of slashes. The new taxa are highlighted in the shaded box. AMA and PHA indicate the presence of amatoxins and phallotoxins, respectively, detected by HPLC. NT indicates not tested.

The test of substitution saturation (Table 2) showed that the observed index of substitution saturation (*Iss*) for the ITS-LSU dataset (ITS and LSU partition considered individually) the *tef-1*, *rpb2*, LSU and β-tubulin alignments of the combined dataset was significantly lower than the corresponding critical index substitution saturation (*Iss.c*), indicating that there was little saturation in our sequences (P < 0.001). On the other hand, the ITS partition of the combined dataset, the *tef-1* introns and the β-tubulin intron showed sign of substitution saturation, indicating the unsuitability of these data for phylogenetic analysis. Nevertheless, re-analysing the ITS-LSU partition with DAMBE, after the exclusion of the 378 sites (40% of a total of 935 sites) retained by Gblocks, the substitution saturation test revealed an Iss value that was significantly (P < 0.001) lower than the Iss.c (Table 2), indicating the suitability of this data for further phylogenetic analysis. We therefore included an ITS partition, excluding the poorly aligned positions identified by Gblocks, in the combined dataset. Regarding the introns partitions, according to Thongbai et al. (2017), *A.zangii* and the *A.ballerina* clade (Fig. 1), should be considered to belong in a different section (*Amanitaincertaesedis*), sister to the *Phalloideae* sensu Bas (1969). We therefore tested the combined dataset for substitution saturation by using *A.zangii* as the outgroup and excluding from the analysis the *A.ballerina* clade and the outgroup. In this case, no sign of saturation was evidenced, which supports the consistency of the phylogenetic signal in the main *Phalloideae* clade. We therefore decided to include the introns partitions in the phylogenetic analyses in order to increase the resolution at species level.

The ITS-LSU dataset and the final combined DNA sequence alignments of all loci (β-tubulin, *rpb2*, ITS, LSU, *tef-1*) alignments contained 15 and 35 OTUs and were 1575 and 3133 sites long including gaps, respectively. Sequence data and statistical analysis for each dataset are provided in Table 2.

**Figure 2.**
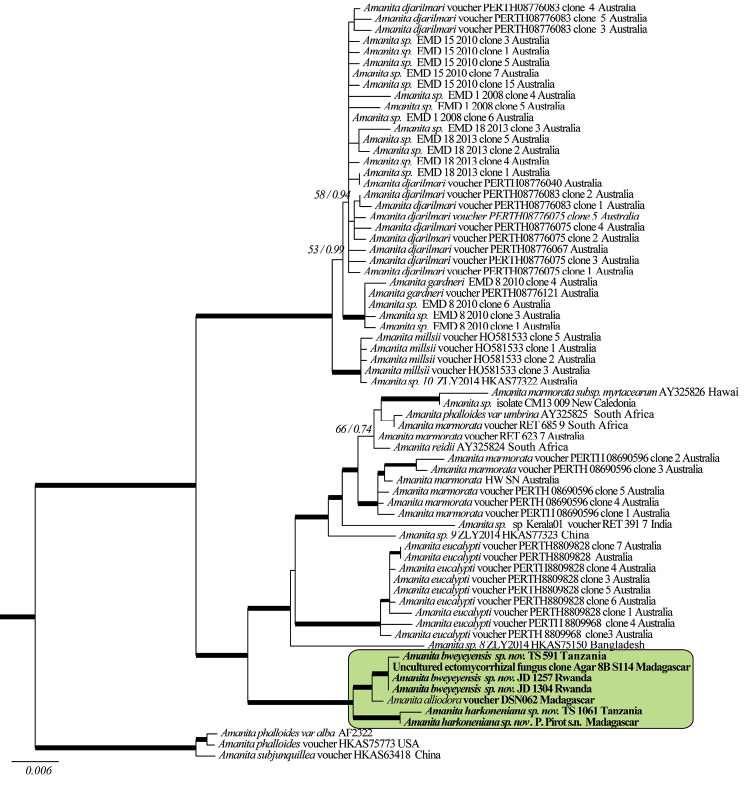
The 50% majority-rule consensus tree from Bayesian inference of the combined nuclear ITS-5.8S and LSU sequences. Thickened branches in bold indicate bootstrap support greater than 70% and Bayesian posterior probability greater than 0.95. For selected nodes, parsimony bootstrap support value and Bayesian posterior probabilities are, respectively, indicated to the left and right of slashes. The new taxa are highlighted in the shaded box.

The topologies obtained by analysing the combined dataset and the ITS-LSU dataset were highly congruent with published trees (Zhang et al. 2010, Cai et al. 2016, Thongbai et al. 2017), at least for what concerns significantly supported branches, and the Bayesian consensus trees (Figs 1 and 2) were almost identical to the optimal trees inferred under the Maximum Likelihood criterion. Several collections from tropical Africa clustered together in a well-supported clade. So far, this clade remains isolated but is notably distantly related to all other *Amanita* species, as yet reported from Africa (Zhang et al. 2010, Cai et al. 2016, Thongbai et al. 2017) or elsewhere and for which sequences are known (Figs 1 and 2), suggesting a common phylogenetic background. *Amanitaalliiodora* clustered together with the two unnamed species from tropical Africa in all phylogenetic inferences considered individually or concatenated (i.e. phylogenetic species, Figs 1 and 2, shaded box).

Morphological examination showed combinations of morphological features unique to and characteristic of each, thereby defining two morphotypes. The critical morphological features that differentiate them are the following. The first species grows under Eucalyptus. Its bulb at stipe base is (sub-)globose, neither pointed nor rooting. The ring is striated and the smell sweetish and conspicuous. The second species is not bound with *Eucalyptus* and has been collected in Miombo woodland and in a garden. The bulb at the stipe base is turnip-shaped to rooting. The ring is smooth or vaguely plicate and the smell weak, resembling raw potato. We therefore concluded that these two morphotypes / clades represent two distinct new species, which we describe below resp. as *A.bweyeyensis* sp. nov. and *A.harkoneniana* sp. nov.

#### PCR amplification of *Amanita* toxins genes family members

By using a combination of the degenerated primers cited above, we obtained a complete 17-mer sequence of phallacidin precursor for the three specimens of *A.bweyeyensis* and the two specimens of *A.harkoneniana* studied (Table 3), comprising the mature toxin region sequence of phallacidin (AWLVDCP) and both the invariant Pro residues immediately preceding the mature peptide sequence and the last amino acid of the toxin. Surprisingly, this is the first time that a complete PHA sequence has been found in a species of Amanitasect.Phalloideae that does not produce this toxin. This finding is in contrast with the study of Hallen et al. (2007), concluding that all of the species synthesising amatoxins and phallotoxins, but none of the other species, hybridised to AMA and PHA genes probes (based on the same primers used in this study). However, while successful PCR amplification proves the presence of a gene (PHA gene in this case), an unsuccessful PCR, possibly due to primer mismatches, cannot be used to prove the absence of the genes encoding α- and β-amanitin, whose exact DNA sequence for these specimens is not known.

**Table 3.** PCR products (phalloidin, PHA gene) amplified from *A.bweyeyensis* and *A.harkoneniana* with degenerate primers, compared to the PHA gene sequences available on GenBank.

						Phallacidin precursor (17-mer)																										
											Phallacidin mature peptide																					
	M	S	D	I	N	A	T	R	L	P	A	W	L	V	D	C	P	C	V	G	D	D	V	N	P	V	L	T	R	G	Q	R
MK570933*A.bweyeyensis* JD 1304	ATG	TCT	GAC	ATC	AAT	GCC	ACC	CGT	CTC	CCT	GCY	TGG	CTT	GTA	GAC	TGC	CCC	TGC	GTC	GGT	GAC	GAC	TGC	AAC	CCC	GTA	CTC	ACT	CGT	GGG	CAG	AGG
MK570932*A.bweyeyensis* JD 1257	ATG	TCT	GAC	ATC	AAT	GCC	ACC	CGT	CTC	CCT	GCY	TGG	CTT	GTA	GAC	TGC	CCC	TGC	GTC	GGT	GAC	GAC	TGC	AAC	CCC	GTA	CTC	ACT	CGT	GGG	CAG	AGG
MK570934*A.bweyeyensis* TS 591	ATG	TCT	GAC	ATC	AAT	GCC	ACC	CGT	CTT	CCT	GCT	TGG	CTT	GTA	GAC	TGC	CCC	TGC	GTC	GGT	GAC	GAC	TGC	AAC	CCC	GTA	CTC	ACT	CGT	GGG	CAG	AGG
MK570936*A.harkoneniana* TS 1061	ATG	TCT	GAC	ATC	AAT	GCC	ACC	CGT	CTT	CCT	GCY	TGG	CTY	GTA	GAY	TGC	CCA	TGC	GTC	GGT	GAC	GAC	TGC	AAC	CCC	GTT	CTC	ACT	CGT	GGG	CAG	AGG
MK570935*A.harkoneniana* P PIROT SN	ATG	TCT	GAC	ATC	AAT	GCC	ACC	CGT	CTT	CCT	GCY	TGG	CTY	GTA	GAY	TGC	CCC	TGC	GTC	GGT	GAC	GAC	TGC	AAC	CCC	GTT	CTC	ACT	CGT	GGG	CAG	AGG
																							V	N	R	L	L	T	R	G	E	S
KF387488 * A. exitialis *	ATG	TCT	GAC	ATC	AAT	GCC	ACC	CGT	CTT	CCT	GCC	TGG	CTC	GTA	GAC	TGC	CCA	TGC	GTC	GGT	GAC	GAC	GTC	AAC	CGC	CTC	CTC	ACT	CGT	GGC	GAG	AGC
																							V	N	R	L	L	T	R	G	E	R
EU196142 * A. bisporigera *	ATG	TCT	GAC	ATC	AAT	GCC	ACC	CGT	CTT	CCT	GCT	TGG	CTT	GTA	GAC	TGC	CCA	TGC	GTC	GGT	GAC	GAC	GTC	AAC	CGT	CTC	CTC	ACT	CGT	GGT	GAG	AGG
																							V	N	F	I	L	T	R	G	Q	K
KF546298 * A. fuligineoides *	???	???	???	???	???	???	???	???	???	CCT	GCT	TGG	CTT	GTA	GAT	TGC	CCA	TGC	GTT	GGT	GAC	GAT	GTC	AAC	TTC	ATC	CTC	ACT	CGT	GGC	CAG	AAG
																							V	N	R	L	L	A	R	G	E	K
KF546296 * A. fuliginea *	???	???	???	???	???	???	???	???	???	CCT	GCT	TGG	CTT	GTA	GAC	TGC	CCA	TGC	GTC	GGT	GAC	GAC	GTT	AAC	CGC	CTC	CTC	GCT	CGT	GGC	GAG	AAG
																							I	N	R	L	L	T	R	G	E	K
KF552098 * A. pallidorosea *	ATG	TCT	GAT	ATT	AAT	GCT	ACG	CGT	CTT	CCC	GCC	TGG	CTT	GTA	GAC	TGC	CCA	TGC	GTC	GGT	GAC	GAC	ATC	AAC	CGC	CTC	CTC	ACT	CGT	GGC	GAG	AAG
KF546303 * A. phalloides *	???	???	???	???	???	???	???	???	???	CCT	GCT	TGG	CTT	GTA	GAT	TGC	CCA	TGC	GTC	GGT	GAC	GAC	ATC	AAC	CGC	CTC	CTC	ACC	CGC	GGC	GAG	AAG
KC778570 * A. oberwinklerana *	???	???	???	???	???	???	???	???	???	CCT	GCT	TGG	CTT	GTA	GAT	TGC	CCA	TGC	GTC	GGT	GAC	GAC	ATC	AAC	CGC	CTC	CTC	ACT	CGT	GGC	GAG	AAG
																							S	N	R	L	L	T	R	G	E	K
KC778568 * A. subjunquillea *	???	???	???	???	???	???	???	???	???	CCT	GCT	TGG	CTT	GTA	GAT	TGC	CCA	TGT	GTC	GGT	GAC	GAC	ATC	AGC	CGC	CTT	CTC	ACT	CGT	GGC	GAG	AAG
KF546306 * A. rimosa *	???	???	???	???	???	???	???	???	???	CCT	GCT	TGG	CTT	GTA	GAC	TGC	CCA	TGT	GTC	GGT	GAC	GAC	ATC	AGC	CGC	CTT	CTC	ACT	CGT	GGC	GAG	AAG

### Taxonomy

#### Amanita bweyeyensis

Taxon classificationFungiAgaricalesAmanitaceae

Fraiture, Raspé & Degreef sp. nov.

MB830175

Figs 3 , 4

##### Diagnosis.

*Amanitabweyeyensis* differs from the closest *Amanita* species by: pileus first pale brownish-grey then entirely whitish or with a faintly yellowish or pale beige shade, basal bulb of the stipe globose, neither pointed nor rooting, basidiospores subglobose to widely ellipsoid (Q = 1.10–1.17–1.28), absence of α- and β-amanitin, phalloidin and phallacidin in its basidiomata, connection with the genus *Eucalyptus* and distribution in Burundi, Rwanda and Tanzania.

##### Holotypus.

RWANDA. Western Prov.: buffer zone Nyungwe forest, Bweyeye (02°36.62'S; 29°14.04'E), ca. 2050 m alt., 16 Apr. 2015, J.Degreef 1304 (BR!).

##### Description.

**Primordium** subglobose, smooth, whitish or with a weak olive tint. **Pileus** 40–73–120 mm diam., first hemispherical then expanding to regularly convex or applanate, without umbo; margin even, not striate nor appendiculate, in some mature specimens the pileipellis does not reach the edge of the pileus, leaving free the extreme tip of the lamellae; first pale brownish-grey (close to 6B2 or 6C2–3), then often entirely whitish or with a faintly yellowish or pale beige shade (between 4A2 and 5B2); somewhat viscid, smooth, devoid of veil remnants. **Lamellae** free, white, becoming slightly yellowish when old and ochraceous, pinkish-beige to pale pinkish-brown on the exsiccates with a narrow white and fluffy edge; mixed with an equal number of lamellulae which are very variable in length and are usually truncated; sub-distant, 8–9 lamellae and lamellulae per cm at 1 cm from the edge of the pileus, about 120–160 lamellae and lamellulae in total (counts on 5 basidiomata), 3–14 mm broad, serrate when seen with a magnifying glass. **Stipe** 65–95–152 × 7–25 mm, ratio length of the stipe/diam. of pileus = 1.04–1.25–1.38; sub-cylindrical, slightly wider just under lamellae, gradually and slightly widened from top to bottom, white, with finely fibrillose surface, hollow (at least on exsiccates). Ring white, hanging, membranous but thin and fragile, finely fibrillose, smooth to somewhat plicate longitudinally, upper part adhering to the stipe and often more or less striate. Basal bulb of the stipe globose, sometimes a bit elongated but neither pointed nor rooting, up to 45 mm wide, surrounded by a white volva (also white inside), membranous, up to 30–35 mm high. **Context** white, soft; smell sweetish, conspicuous; taste not recorded.

**Basidiospores** hyaline, with thin, amyloid wall, (globose-) subglobose to widely ellipsoid (-ellipsoid), rather often with a mangiform or amygdaliform profile, (7.5-) 8.0–8.81–9.5 (-11.0) × (6.0-) 7.0–7.54–8.5 (-9.0) µm, Q = (1.00-) 1.10–1.17–1.28 (-1.58) [112/4/2]. **Basidia** 4-spored, without clamp, thin-walled, clavate, often rather abruptly swollen, 36–42.3–50 × (8.0-) 10.5–12.0–14 (-15) µm, l/w = 2.6–3.59–4.2 (-5.5) [66/4/2]. **Lamellar edge** sterile, composed of sphaeropedunculate marginal cells which are widely clavate to pyriform, hyaline, thin-walled, smooth, without clamp, 18–26.3–32 (-37) × 12–17.0–20 (-33) µm, l/w = (1.00-) 1.33–1.57–1.83 (-2.33) [40/4/2]. **General veil** (volva) mostly composed of cylindrical hyphae, with very different diameters, (15-) 35–80 (-110) × 2–8.5–15 (-26) µm, hyaline, with smooth and thin wall, septate, with rather frequent anastomoses between parallel hyphae, without clamps, branched, mixed with very few sphaerocysts, thin-walled, smooth, globose to ovoid, 33–76–125 × (25-) 32–56–95 µm, l/w = 1.00–1.52–2.25 [20/2/2].

**Figure 3.**
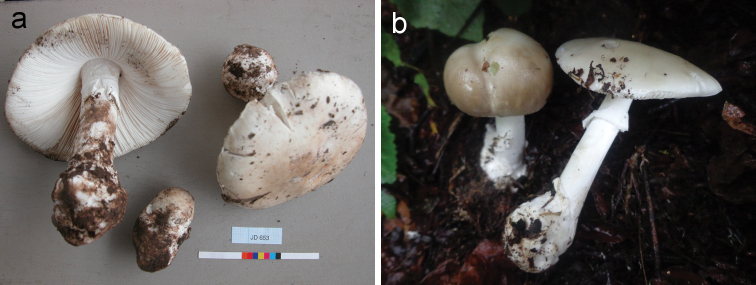
Basidiomata of *Amanitabweyeyensis*. **a** Degreef 653 **b** Degreef 1257.

**Figure 4.**
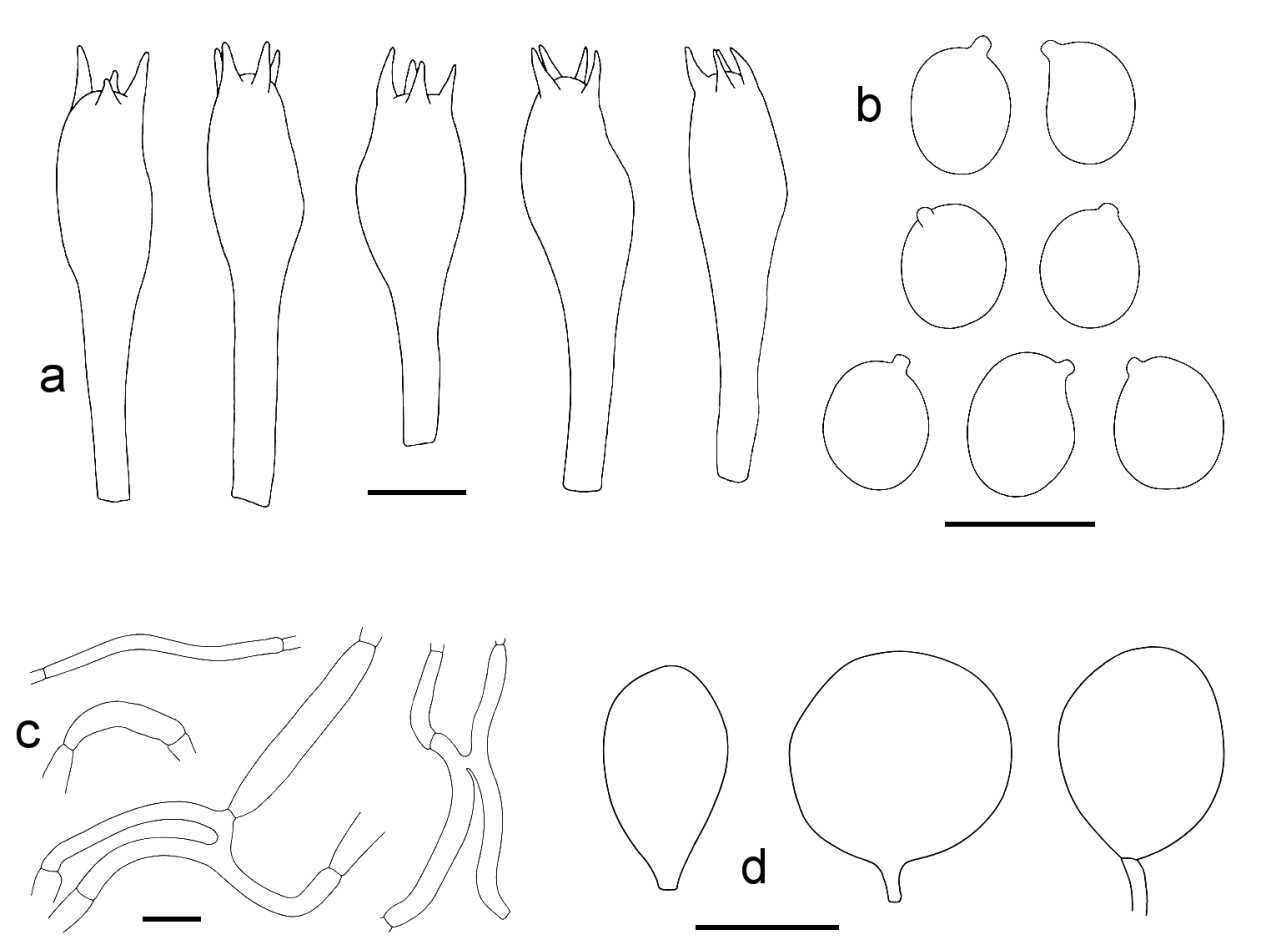
*Amanitabweyeyensis***a** Basidia (from Degreef 1257, scale bar: 10 µm) **b** Spores (from Saarimäki et al. 591, scale bar: 10 µm) **c** Filamentous hyphae from the volva (from Degreef 1304, holotypus, scale bar: 20 µm) **d** Sphaerocysts from the volva (from Degreef 1304, holotypus, scale bar: 50 µm).

##### Distribution.

At present, the species is only known from Burundi, Rwanda and Tanzania but, according to its ecology, it could probably be observed in all *Eucalyptus* plantations in tropical Africa and possibly in South Africa as well. Consequently, if the species is collected for consumption, care should be taken to avoid confusion with *A.marmorata*, a species growing in the same biotopes and suspected to be highly toxic.

##### Ecology.

On the ground, under *Eucalyptus*. The label of *Saarimäki* 591 indicates “in *Acacia* and *Eucalyptus* forest” whereas the legend of the associated picture (Härkönen et al. 2003: 62) indicates “growing in an *Acaciamearnsii* plantation”. However, the litter visible on that picture does not correspond to the latter species but looks like *Eucalyptus* leaves.

##### Etymology.

This species is named after the collection locality of the type specimen in Rwanda.

##### Specimens examined.

BURUNDI. Muravya Prov.: Bugarama, 9 Jan. 2011, J.Degreef 653 (BR). – RWANDA. Western Prov.: buffer zone Nyungwe forest, Bweyeye (02°36.79'S; 29°14.01'E), ca. 2040 m alt., 20 Oct. 2014, J.Degreef 1257 (BR); Ibidem (02°36.62'S; 29°14.04'E), ca. 2050 m alt., 16 Apr. 2015, J.Degreef 1304 (holotype: BR!). – TANZANIA. Pare District: South Pare Mts., Mpepera, ca. 1600 m alt., 5 Dec. 1990, T.Saarimäki et al. 591 (H).

##### Notes.

During collecting field trips in Rwanda, one of us (JD) was confused by observing local people (Abasangwabutaka) picking huge quantities of this mushroom in old *Eucalyptus* plantations and eating them (after removal of the cuticle) without experiencing any trouble. The species was not observed to be eaten in Burundi and is probably not used in Tanzania either.

It is quite likely that the specimen shown in a picture by van der Westhuizen and Eicker (1994: 38) under Amanitaphalloidesvar.alba is *Amanitabweyeyensis*. This specimen was observed at Sabie (South Africa), growing in the leaf-litter under *Eucalyptuscloeziana* in early December and again in March. The pileus surface is described as “white and occasionally faintly yellowish over the central part” and the pileus margin as “very finely denticulate”. Härkönen et al. (2003: 62) already drew attention to that picture.

A comparison with the closely related species is given in the chapter “discussion” below.

#### Amanita harkoneniana

Taxon classificationFungiAgaricalesAmanitaceae

Fraiture & Saarimäki sp. nov.

MB830176

Figs 5 , 6

##### Diagnosis.

*Amanitaharkoneniana* differs from the closest *Amanita* species by: pileus first whitish to pale yellowish-beige then entirely whitish, devoid of veil remnants, basal bulb of the stipe turnip-shaped or irregularly elongated and more or less rooting, basidiospores subglobose to widely ellipsoid (Q = 1.04–1.13–1.25), basidia 34–37.5–41 µm long and growth without connection with the genus *Eucalyptus*, in Tanzania and Madagascar.

##### Holotypus.

TANZANIA. Tabora District: ca. 10 km S of Tabora, Kipalapala, ca. 1200 m alt., 12 Dec. 1991, T.Saarimäki et al. 1061 (H!).

##### Description.

**Primordium** smooth, subglobose but with a more or less conical or irregular rooting part; veil whitish; pileus with a weak brownish tint (around 4B2–3 and 5B2–3 but paler). **Pileus** 35–53–70 mm diam., first hemispherical, then largely conical or convex to nearly applanate, often with a deflexed margin, without umbo; margin even, neither striate (sometimes striate on exsiccates) nor appendiculate; first whitish to pale yellowish-beige (between 4A2 and 4B2) then entirely whitish; slightly viscid when young, smooth, devoid of veil remnants. **Lamellae** white, becoming slightly yellowish when old and pale to dark brownish in exsiccates with a narrow white and fluffy edge, free, mixed with an equal number of lamellulae which are very variable in length and are usually truncated, sub-distant, 8–10 lamellae and lamellulae per cm at 1 cm from the edge of the pileus, about 125–215 lamellae + lamellulae in total (counts on 2 basidiomata), ventricose, very finely serrate when seen with a magnifying glass. **Stipe** 65–130 × 8–14 mm, sub-cylindrical, slightly wider just under the lamellae, gradually and slightly widened from top to bottom, white, with finely fibrillose surface, hollow (at least in exsiccates) or stuffed. Ring white, hanging, membranous but thin and fragile, upper part adhering to the stipe. Basal bulb of the stipe turnip-shaped or irregularly elongated, more or less rooting, surrounded by a white volva (also white inside), membranous, up to 40–60 mm high. **Context** white, soft, very thin along the margin of the pileus, much thicker near the stipe; smell weak resembling raw potato [Harkonen pers. comm.], very variable according to specimens but mostly of shellfish as in *Russulaxerampelina*, especially for mature and old specimens [P. Pirot, pers. comm. about specimens from Madagascar], taste mild, then unpleasant [description of the Tanzanian specimen].

**Figure 5.**
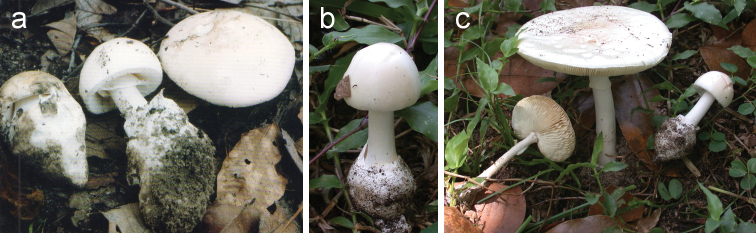
Basidiomata of *Amanitaharkoneniana***a** Saarimäki et al. 1061 (holotypus) **b** Pirot s.n. (coll. 2014) **c** Pirot s.n. (coll. 2014).

**Figure 6.**
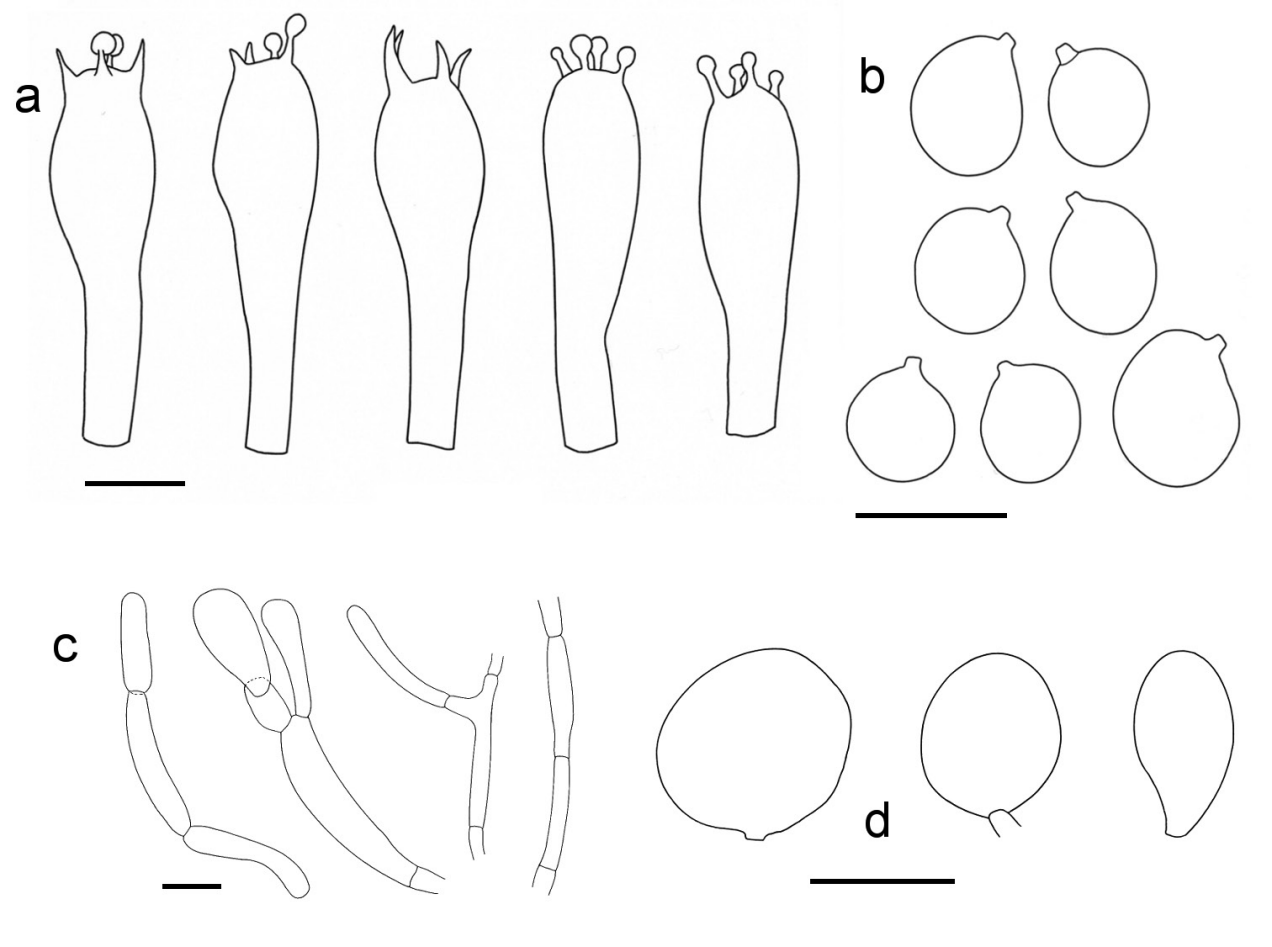
*Amanitaharkoneniana* (all from Saarimäki et al. 1061, holotypus) **a** Basidia (scale bar: 10 µm) **b** Spores (scale bar: 10 µm) **c** Filamentous hyphae from the volva (scale bar: 20 µm) **d** Sphaerocysts from the volva (scale bar: 50 µm).

**Basidiospores** hyaline, with thin, rather weakly amyloid wall, (globose-) subglobose to widely ellipsoid (-ellipsoid), (6.5-) 7.0–8.07–8.6 (-10.0) × (6.0-) 6.5–7.15–8.0 (-8.5) µm, Q = (1.00-) 1.04–1.13–1.25 (-1.33) [53/3/2]. **Basidia** 4-spored, without clamp, clavate, often rather abruptly swollen, (30-) 34–37.5–41 (-46) × 9.0–10.4–11.0 (-13.0) µm, l/w = 3.00–3.60–4.40 (-4.90) [31/3/2]. **Lamellar edge** sterile, composed of marginal cells which are widely clavate to pyriform, hyaline, thin-walled, smooth, not clamped, 26–32.2–40 × 13–16.8–20 µm, l/w = 1.56–1.93–2.23 [10/1/1]. **General veil** (volva) mostly composed of cylindrical hyphae, with very different diameters, (20-) 33–50 (-110) × 4–11 (-15) µm, hyaline, with smooth and thin wall, septate but without clamps, with occasional anastomoses between parallel hyphae, branched, mixed with a few scattered hyaline sphaerocysts, globose to sphaeropedunculate or ellipsoid, 45–75–100 (-120) × (20-) 35–57–87 (-115) µm, l/w = 1.04–1.38–1.68 (-2.38), with a smooth and thin wall, rarely slightly thickened (< 1 µm) [18/1/1].

##### Distribution.

Up to now, the species is only known from Tanzania and Madagascar. According to its ecology, it could potentially be observed in all regions occupied by the miombo woodland.

##### Ecology.

In miombo woodland (Tanzania) and in a garden, next to *Cocosnucifera* L., *Citrus* sp. (“combava”), *Tambourissa* sp. and *Psidiumguajava* L., along the Indian Ocean (Madagascar).

##### Etymology.

This species is dedicated to Prof. Marja Härkönen in acknowledgment of her tremendous contribution to African mycology.

##### Specimens examined.

MADAGASCAR. Prov. Toamasina: Mahambo, Dec. 2014, P.Pirot s.n. (BR); Ibidem, 2016, P.Pirot s.n. (BR). – TANZANIA. Tabora District: ca. 10 km S of Tabora, Kipalapala, ca. 1200 m alt., 12 Dec. 1991, T.Saarimäki et al. 1061 (holotype: H!).

##### Note.

We believe that the picture of “*Amanita* cfr. *phalloides*” presented by Ryvarden et al. (1994: 76–77) could be *Amanitaharkoneniana*. The macroscopic description and the picture given by the authors correspond to the characters of that species. From this description, the fruit-bodies have a nauseous odour, are soon decaying and grow in miombo woodlands or in association with pine trees in the middle of the rainy season; they are rarely seen. No precise locality is given but the book covers South Central Africa (mostly Malawi, Zambia and Zimbabwe).

A comparison with the closely related species is given in the chapter “discussion” below.

### Chemical analyses

RP-HPLC analyses of the specimen Degreef *1304* (holotypus of *A.bweyeyensis*) was made by two of us (EK & IA). The analysis showed the complete absence of α-, β- and γ-amanitin as well as that of phallacidin and phalloidin. The results were below the limit of detection (0.6 ng/g) for all the toxins in all the analysed samples: 3 samples with cuticle and 3 samples without cuticle.

It is interesting to mention that another specimen of *A.bweyeyensis* (*Tiina Saarimäki et al.* 591), collected in Tanzania, had been analysed previously, in the Technical Research Centre of Finland in Espoo, and that neither amatoxins nor phallotoxins had been found in that specimen either (Harkonen pers. comm.).

### Identification key to the African and Madagascan species of Amanitasect.Phalloideae

**Table:** 

1	Spores elongated, Q > 1.45. Slender species, ratio stipe length / pileus diameter > 1.5. Ring funnel-shaped on young basidiomata, not striated. Pileus margin often striated because of the thinness of the flesh	***Amanitastrophiolata*** [incl. var. bingensis]
	Pileus 50–60 mm diam., dirty white, often with a yellowish or greenish centre. The original description of var. bingensis mentions a pungent taste. Spores (7-) 7.5–10.0 (-10.5) × (4.0-) 5.0–6.5 (-7.0) µm, Q = 1.40–1.75.	
–	Spores less elongated, Q < 1.45. Less slender species, ratio stipe length / pileus diameter < 1.5. Ring never ascending, striated or not. Pileus margin not striated	**2**
2	Pileus greenish or olivaceous, sometimes yellowish-green or brownish-green, virgate (i.e. with fine darker radial stripes). Smell of old rose or rotten honey in age	*** Amanita phalloides ***
	Pileus 65–152 mm diam., ring striate. Spores 7.5–10.0 (-12.5) × (5.5-) 6.0–7.5 (-8.0) µm.	
–	Pileus whitish, greyish or pale brownish (sometimes olivaceous grey with a paler margin but then, strong smell of garlic), not virgate but sometimes radially marbled. Smell fungoid or different	**3**
3	Strong garlic smell, persisting several months in herbarium specimens. Spores subglobose, mean Q < 1.5	*** Amanita alliiodora ***
	Pileus viscid, olivaceous grey, with a pallid margin, about 50 mm diam., ring striated.	
–	Smell fungoid or different. Spores subglobose or more elongated	**4**
4	Lamellae staining yellowish when bruised	*** Amanita thejoleuca ***
	Pileus 60–80 mm diam., pale yellowish-brown, darker in the centre. Ring rather fugacious, often missing on mature specimens. Spores 7–8 × 5–6 µm (original description), or 10–12 × 7.5–10 µm (after the spore drawings in Gilbert, 1941)	
–	Gills not yellowing when bruised	**5**
5	Pileus white at first, soon radially marbled by pale brownish or greyish streaks. Species mostly associated with various species of *Eucalyptus*, also mentioned once under *Casuarinaequisetifolia*	*** Amanita marmorata ***
	Pileus 25–95 mm diam., ring striated. Spores (6.5-) 7.5–9.5 (-11.5) × (5.5-) 6.0–8.0 (-10.0) µm, Q = 1.05–1.40	
–	Pileus not marbled, uniformly coloured or paler at margin, whitish to mouse grey or pale brownish. Species bound or not with *Eucalyptus*	**6**
6	Pileus mouse grey, dry. Ring striated	*** Amanita murinacea ***
	Pileus 70–80 mm diam. Spores 7.5–8.5 × 7–8 µm, mean Q = 1.15	
–	Pileus whitish to pale brownish or greyish, often more or less viscid. Ring striated or not	**7**
7	Species growing under *Eucalyptus*. Bulb at stipe base +/- globose, neither pointed nor rooting. Ring striated. Smell sweetish, conspicuous	*** Amanita bweyeyensis ***
–	Species not bound with *Eucalyptus*, found in Miombo woodland and in a garden. Bulb at stipe base turnip-shaped to rooting. Ring smooth or vaguely plicate. Smell weak resembling raw potato	*** Amanita harkoneniana ***

## Discussion

The fact that *A.bweyeyensis* seems to grow always in association with *Eucalyptus* species (Myrtaceae), which are not indigenous in Africa, suggests that the fungus has been introduced with the trees. Such introductions are well known (see for example Díez 2005). Vellinga et al. (2009) stress the fact that Pinaceae and Myrtaceae are the plant families which are the most frequently reported as hosts of introduced mycorrhizal fungi. They also mention that South Africa is the African country with the highest number of mycorrhizal introductions. We therefore compared *A.bweyeyensis* more specifically with the Australian species of Amanitasect.Phalloideae (Reid 1979, Miller 1991, Wood 1997, Davison et al. 2017, Tulloss 2018). We believe that conspecificity with any of these species can be excluded, because they present one or several of the following characters: spores too elongated (mean Q ≥ 1.4), pileus strongly coloured (brown or grey), pileus with patches of general veil, ring absent, stipe not bulbous, different host, toxin content etc.

*Amanitamarmorata* Cleland & E.-J. Gilbert was described from New South Wales (Australia) (Gilbert 1941). It was subsequently re-described from South Africa, under the name *A.reidii* Eicker & Greuning (Eicker et al. 1993, Cai et al. 2014) and from Hawaii, sub A.marmoratasubsp.myrtacearum O.K. Mill., Hemmes & G. Wong (Miller et al. 1996). Before the description of *A.reidii* in 1993, African collections of that taxon were often called Amanitaphalloidesvar. orf.umbrina (see e.g. van der Westhuizen and Eicker 1994:41). The species is present in Africa and it grows in connection with the genus *Eucalyptus* but it can be separated from *A.bweyeyensis* by its whitish pileus marbled with grey brown radial streaks and by the presence of phalloidin and phallacidin in its basidiomata (Hallen et al. 2002, Davison et al. 2017). The presence of α- and β-amanitin in *A.marmorata* remains ambiguous. Hallen et al. (2002) stated that those toxins were present in the species (sub *A.reidii* and probably also sub A.phalloidesf.umbrina), whilst Davison et al. (2017) could not detect them. The marbled colour, the globose bulb and the connection with *Eucalyptus* also exclude conspecificity with *A.harkoneniana*. *A.marmorata* is also well separated from our two new species in all the phylogenetic inferences (Figs 1 and 2).

Three new species of *Phalloideae* were recently found in Australia, namely *Amanitadjarilmari* E.M.Davison and *A.gardneri* E.M.Davison from the south-west of Australia and *A.millsii* E.M.Davison & G.M.Gates from Tasmania (Davison et al. 2017). The three species have a white- or pale-coloured pileus and a white universal veil. They are quite similar to our two new species, but are however well separated from them in the phylogenetic trees (Figs 1 and 2). The following differences with *A.bweyeyensis* can also be cited: *A.djarilmari* has elongated spores (mean Q = 1.43) and contains phallacidin and phalloidin; *A.gardneri* has a fusiform bulb at stem base, becoming radicant, very elongated spores (mean Q = 1.81) and contains phallacidin and phalloidin; *A.millsii* is apparently not connected with *Eucalyptus* species and it contains phallacidin and phalloidin. The three species can be separated from *A.harkoneniana* by the following characters: *A.djarilmari* has a rounded bulb at stem base and elongated spores (mean Q = 1.43); *A.gardneri* has very elongated spores (mean Q = 1.81); *A.millsii* shows persistent patches of universal veil on the pileus, its basidiomata have a more squat habit and its basidia are longer (43–61 µm).

*Amanitacapensis* A. Pearson & Stephens is a nom. nud. which was published in Stephens and Kidd (1953) and quite largely used in South Africa (“Cape death cap”). It is usually considered as a stouter colour variant of *Amanitaphalloides*, including specimens with a whitish pileus (Levin et al. 1985, Reid and Eicker 1991). Several cases of severe poisoning have been attributed to the “species”, some of them fatal (Stephens and Kidd 1953, Steyn et al. 1956, Sapeika et al. 1960). It is therefore surprising that Hallen et al. (2002) did not find the toxins in a specimen identified as *A.capensis* but the exact identity of the fungus remains uncertain and confusion with another taxon cannot be excluded. Conspecificity with *A.bweyeyensis* can be rejected amongst others because of the toxicity and pileus colour of *A.capensis* as well as of its association with other trees than *Eucalyptus*. *Amanitacapensis* differs from *A.harkoneniana* amongst others because it has a larger size than this latter species, a globose bulb and a striated ring.

*Amanitaalliiodora* Pat. is a very poorly known species, described from Madagascar by Patouillard (1924, corrected version in 1928). The most important characteristics of the species are the following. Pileipellis pale olivaceous grey, whitish at the margin, viscid when moist. Ring striated. Spores subglobose, 7–8 µm diam. (Patouillard 1924, 1928) or 8–8.5 µm diam. (Dujarric de la Rivière and Heim 1938), or 8.5–9.6 × 7.8–8.7 µm (Tulloss 2017, after the spore drawings from the type specimen, published in Gilbert 1941). The species is also said to have a bitter taste and to produce a strong smell of garlic, still persisting on exsiccates. It is considered toxic and is not eaten by the local population, which however uses its odour to cure headaches. *A.alliiodora* is distinct from *A.bweyeyensis* because it has a grey pileus and a strong smell of garlic and also because it does not grow in association with *Eucalyptus* and is probably toxic. It is also distinct from *A.harkoneniana* because of the grey pileus, the striated ring and the smell of garlic. *A.alliodora* clustered in a sister position to *A.bweyeyensis* in the ITS-nucLSU based phylogenetic analysis, forming a two-species clade sister to *A.harkoneniana*, showing that these three species share a common phylogenetic background.

Within the genus *Amanita*, the genes encoding amatoxins (α- and β-amanitin) and phallotoxins (phallacidin and phalloidin) were found so far to be present only in species that produce these compounds (Hallen et al. 2007). The successful PCR amplification of the PHA gene for both *A.bweyeyensis*, a species which is regularly consumed by local people and *A.harkoneniana* was indeed surprising. Especially since the HPLC analysis did not show any sign of these compounds in the basidiomata. This is the first time that the presence of at least one of those genes (PHA gene) could be proven for species that seem to lack (or have lost) the ability to produce these toxins.

Very little is indeed known about the mechanisms behind the regulation of the fungal secondary metabolism. Many factors can play a key role in preventing the expression of phallacidin gene in these species. Several studies (Enjalbert et al. 1993, 1999; Brüggemann et al. 1996; Mcknight et al. 2010; Kaya et al. 2015) have shown that phallotoxin amounts and distribution (localisation in the basidiome) in *A.phalloides* largely vary as a result of environmental and climatic conditions. Furthermore, several studies have shown that the toxin concentration in the pure cultured mycelium of deadly *Amanita* is about 10% of that in basidiomata and that it is indeed possible to increase the amatoxin production through optimisation of growth conditions, such as medium composition, pH and temperature etc. (Zhang et al. 2005, Hu et al. 2012). Furthermore, temporal and structural sequestration of secondary metabolites are common features in microorganisms. Amatoxins and phallotoxins are biologically active secondary metabolites and some mechanism of separation from primary metabolism seems to be essential to avoid their coming into contact with their sites of action (RNA polymerase II and F-actin, respectively). Having higher toxin concentrations only in the basidiome, or part of it, would invest resources for defence where it is especially needed, in the visible and vulnerable mushroom and not microscopic spores or mycelia.

Amatoxins and phallotoxins are encoded by members of the “MSDIN” gene family and are synthesised on ribosomes as short (34- to 35-mer) pro-proteins, with conserved upstream and downstream sequences flanking a hypervariable region of 7 to 10 amino acids (Hallen et al. 2007, Luo et al. 2012). The hypervariable region gives rise to the linear peptides corresponding to the mature toxins. The precursor peptides must undergo several post-translational modifications, including proteolytic cleavage, cyclisation, hydroxylation and formation of a unique tryptophan-cysteine cross bridge called tryptathionine. In particular, they are cleaved and macrocyclised into 7–10 amino acid cyclic peptides by a specialised prolyl-oligo-peptidase enzyme (POP), which is the key enzyme of the cyclic peptide pathway, catalysing both hydrolysis (Luo et al. 2009, 2014; Riley et al. 2014).

The genes of most secondary metabolite biosynthetic pathways tend to be clustered and co-regulated in fungi (e.g. fumonisin biosynthesis in *Fusarium*). Many, but not all, clusters contain cluster-specific transcription factors that regulate expression of the biosynthetic genes for their respective metabolites, thus allowing for multiple regulatory layers giving the producing fungus precise spatial and temporal control over metabolite expression. A mutation in each key protein involved in the biosynthetic/regulatory pathway of phallotoxins production could result in an altered expression of the toxin. The evolutionary persistence of toxins productions in AmanitasectPhalloideae suggests that it should confer some selective advantage to the producing fungi. Since the lack of toxins could be the result of an alteration of the expression of these genes due to environmental and climatic conditions, in our opinion *A.bweyeyensis* and *A.harkoneniana* should be considered to have the potential to be deadly poisonous.

## Supplementary Material

XML Treatment for Amanita bweyeyensis

XML Treatment for Amanita harkoneniana
